# The scent of mixtures: rules of odour processing in ants

**DOI:** 10.1038/srep08659

**Published:** 2015-03-02

**Authors:** Margot Perez, Martin Giurfa, Patrizia d'Ettorre

**Affiliations:** 1 Research Center on Animal Cognition; University of Toulouse; UPS; 118 route de Narbonne, F-31062 Toulouse Cedex 9, France; 2 Research Center on Animal Cognition; CNRS; 118 route de Narbonne, F-31062 Toulouse Cedex 9, France; 3 Laboratory of Experimental and Comparative Ethology, University Paris 13, Sorbonne Paris Cité, Villetaneuse, France

## Abstract

Natural odours are complex blends of numerous components. Understanding how animals perceive odour mixtures is central to multiple disciplines. Here we focused on carpenter ants, which rely on odours in various behavioural contexts. We studied overshadowing, a phenomenon that occurs when animals having learnt a binary mixture respond less to one component than to the other, and less than when this component was learnt alone. Ants were trained individually with alcohols and aldehydes varying in carbon-chain length, either as single odours or binary mixtures. They were then tested with the mixture and the components. Overshadowing resulted from the interaction between chain length and functional group: alcohols overshadowed aldehydes, and longer chain lengths overshadowed shorter ones; yet, combinations of these factors could cancel each other and suppress overshadowing. Our results show how ants treat binary olfactory mixtures and set the basis for predictive analyses of odour perception in insects.

Odours play a key role in most aspects of animal behaviour. In nature, animals are usually confronted with complex olfactory mixtures, which are composed of numerous components. Thus, the question of how animals perceive and learn odour mixtures is the subject of current debates in fields as diverse as neurosciences^1^, experimental psychology^2^, animal behaviour^3^, computational biology^4^ and chemical ecology^5^, among others. Animals, such as insects, for which odour cues play a significant role in their natural life, constitute suitable models for the study of olfactory mixture perception. In addition, the existence of protocols for olfactory conditioning in these animals allows uncovering the mechanisms underlying the processing of complex odour blends.

A phenomenon particularly useful for studying how animals perceive stimulus mixtures is overshadowing^6^,^7^,^8^, which occurs when two criteria are fulfilled: 1) an individual trained with a binary mixture responds less to one component (‘overshadowed component’) than to the other (‘overshadowing component’); 2) the response to the overshadowed component is lower than that obtained when this component is trained alone. The occurrence of overshadowing therefore reveals that sometimes animals do not respond to all mixture components, but that the most salient component dominates and drives the animal's responses^9^,^10^,^11^.

Olfactory overshadowing has been shown in a variety of taxa, including mammals^12^, crustaceans^13^ and insects^14^,^15^,^16^,^17^,^18^ but the rules underlying this phenomenon remain unclear due to the lack of systematic analyses. With their relatively simple brains, insects are valuable models for the study of olfactory perception and learning, both at the behavioural and neurophysiological level^19^,^20^. Among insects, the honeybee *Apis mellifera* has been extensively used to this end^21^ but the rules and mechanisms of olfactory overshadowing are still unclear in this insect^18^. Ants constitute an interesting model for the study of odour learning and perception, as they are typically confronted to odour mixtures in their natural life (e.g. floral odours when foraging, signature mixtures in nestmate recognition)^22^. Due to the varieties of their life-history traits and ecological adaptations, ants have gained increased popularity in recent behavioural and neurobiological olfactory research^23^,^24^,^25^,^26^,^27^,^28^,^29^,^30^,^31^,^32^; for instance, controlled olfactory appetitive learning protocols have been established for these insects^24^,^26^,^29^. As a consequence, the study of perception and learning of odour mixtures is timely and biologically relevant in this taxon.

Here, we aimed at uncovering the rules accounting for olfactory overshadowing in the ant *Camponotus aethiops*, an insect which offers the advantage of being easily conditionable using an appetitive protocol in which it learns to associate an odorant with a reward of sucrose solution^26^. Ants having learnt the association exhibit the appetitive *maxilla-labium* extension response (MaLER) to the learnt odorant (the conditioned stimulus, or CS). We trained *C. aethiops* ants with odours that are part of floral blends^33^ and that are thus relevant for this species, which forages partly on extra-floral nectaries. Conditioned odours were either single odours or binary mixtures of these odours. Four alcohols and four aldehydes, which varied systematically in their carbon-chain length, were arranged in two sets (6 vs. 8 carbons, and 7 vs. 9 carbons) in order to allow similar comparisons between components in terms of functional group and carbon-chain length, two physical dimensions that define a putative olfactory space in insects^34^. By equilibrating the vapour pressure of all odorants, we aimed at controlling for concentration effects^12^,^35^, thus focusing only on functional group and carbon-chain length effects. We show that olfactory overshadowing resulted from the interaction between chain length and functional group. Generally, alcohols overshadowed aldehydes, and longer chain lengths overshadowed shorter ones; yet, a combination of these dimensions could cancel each other and suppress overshadowing. The emergence of these similar, reproducible dominance patterns in the two sets of odours argues in favour of predictive rules based on the chemical proprieties of odours.

## Results

### Ants successfully learnt and memorized all odour stimuli

In the first experiment, three groups of ants were conditioned in parallel for each combination of two odours, A and B: one group was conditioned to the binary mixture AB (*OVS* group) and the other two to only one of the odour components (A for *Ctrl A* group; B for *Ctrl B* group). In all cases, ants were conditioned along 6 spaced trials. Two sets of four odours (set 1: hexanol, hexanal, octanol, octanal; set 2: heptanol, heptanal, nonanol, nonanal) were used to establish 12 binary mixtures (6 by set of odours). Within each set, odorant molecules varied in their functional group (aldehyde or alcohol) and carbon number (6 vs 8 carbons for set 1; 7 vs 9 carbons for set 2). In total, 1060 ants were conditioned in this experiment.

**Figure 1:**
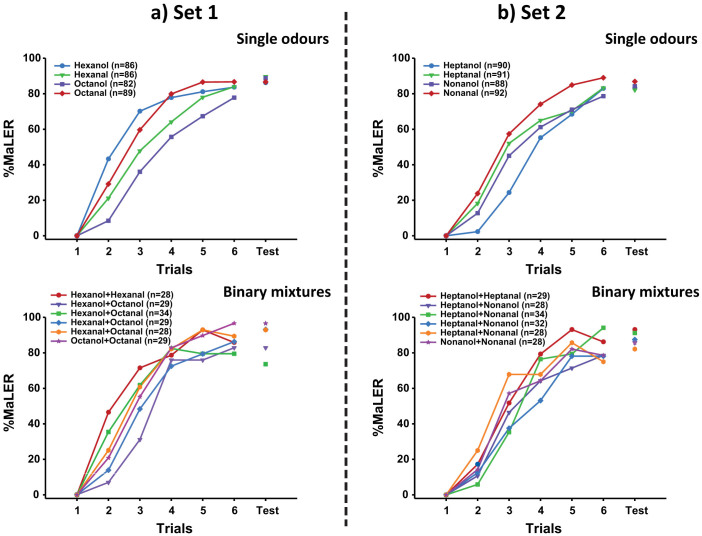
Ants successfully learnt all single odours and binary mixtures. Learning curves of single odours and binary mixtures for odour set 1 (a) and odour set 2 (b). The graphs show the percentage of ants responding to the conditioned odour with the MaLER during six successive conditioning trials. Sample size (n) for each odour is indicated in parentheses. For single odours, curves represent the pooled performance of all groups in which a given odour was used as CS in experiment 1 (for more details about statistics, see Supplementary Information). For all single odours and binary mixtures of each odour set, ants reached the same level of conditioned responses in the last conditioning trial and their performance did not decay 1 h after training, as revealed by the memory test (shown as separate plot in the same graphs).

Irrespectively of using single odours or binary mixtures as CS, ants efficiently learnt the odour-sucrose association and exhibited a high level (approximately 85%) of conditioned responses at the end of training (Fig. 1). Within each odour set, single odours and binary mixtures were learnt equally well, as revealed by similar levels of conditioned responses in the last trial (Fig. 1a: set 1, single odours: χ^2^ = 2.25; p = 0.52, binary mixtures: χ^2^ = 3.92; p = 0.56; Fig. 1b: set 2, single odours: χ^2^ = 1.07; p = 0.78, binary mixtures: χ^2^ = 4.82; p = 0.44). Furthermore, we neither found significant differences in the level of conditioned responses between single odours and mixtures (Fig. 1a: set 1, χ^2^ = 1.15; p = 0.28; Fig. 1b: set 2, χ^2^ = 0.07; p = 0.80) nor between sets of odours (Fig. 1a, b, single odours: χ^2^ = 0.06; p = 0.81; binary mixtures: χ^2^ = 1.59; p = 0.21).

One hour after training, all groups were tested without reinforcement with the mixture AB, each of the odour components (A and B) and a novel odour C, which was unknown to the ants and therefore allowed to measure the tendency to generalise olfactory responses. Odour presentation was randomized in the test. To verify that performances in the memory test did not decay after training, we compared the level of conditioned responses of the learnt odorant in the test with that obtained in the last conditioning trial. Within each odour set, responses in the last conditioning trial did not differ from those recorded in the memory test (Fig. 1a: set 1, single odours: 0 < χ^2^ < 0.5; 0.48 < p < 1, binary mixtures: 0 < χ^2^ < 0.5; 0.48 < p < 1; Fig. 1b: set 2, single odours: 0.08 < χ^2^ < 0.57; 0.45 < p < 0.77, binary mixtures: 0.17 < χ^2^ < 1.33; 0.24 < p < 0.68), except for octanol for which responses even increased (χ^2^ = 4.27; p < 0.05).

Altogether, these results show that ants successfully learn odour-sucrose associations, both in the case of single odours and binary mixtures, and that the information learnt is efficiently retained one hour after conditioning.

### The ants' response to test odours revealed the occurrence of overshadowing

**Figure 2:**
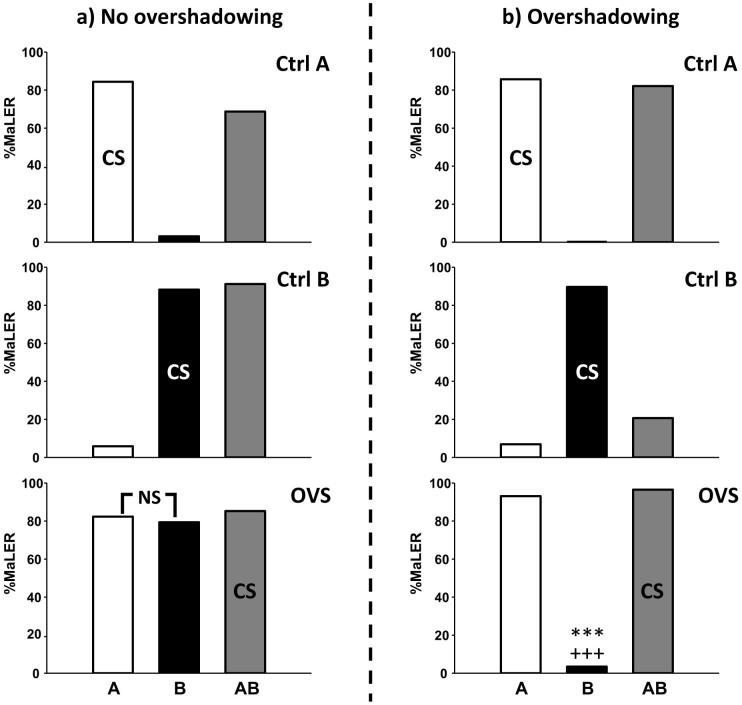
The case of overshadowing. Three groups of animals are trained independently, two with the single odours A and B (*Ctrl A* and *Ctrl B*, respectively), and one with the binary mixture AB (*OVS*) as conditioned stimulus (CS). The figure shows the percentage of ants responding to the conditioned odour with MaLER in a test performed 1 h after training. Each group was tested with A (white bar), B (black bar) and AB (grey bar). All groups responded highly to their respective CS. (a) Example of absence of overshadowing: the *OVS* group responds equally to both components A and B of the mixture (example from the heptanol/nonanal combination, see Supplementary Fig. 3b). (b) Example of overshadowing: the *OVS* group responds significantly less to B than A (***) and responds also significantly less to B than after training to B alone (+++; see black bars of *Ctrl B* and *OVS*) (example from the octanol/octanal combination, see Supplementary Fig. 6a). (NS) non-significant.

During the tests, ants trained with a binary mixture AB could in principle respond to A and B with the same level of responses reached by these components when trained alone, thus revealing an absence of overshadowing. Figure 2a shows an example in which the group trained with the mixture (*OVS*) responds to the components A and B in a similar way, and with levels undistinguishable from those of the groups trained to the single components (*Ctrl A* and *Ctrl B*). Alternatively, ants could respond significantly less to one component than to the other (e.g. B < A), and less to this component than when it was learnt alone (the two criteria for defining overshadowing, see Introduction). Figure 2b shows an example in which overshadowing occurs as the response to component B of the *OVS* group is not only inferior to A but it is also inferior to the response to B of the *Ctrl B* group.

The 12 binary odour combinations of alcohols and aldehydes of sets 1 and set 2 are shown in Supplementary Table 1, together with the ketone used as novel odour for a generalisation control. The complete results obtained for each odour combination are shown in Supplementary Figs. 1–6. In all cases, the response to this ketone was low, thus showing a low generalization from the trained alcohols or aldehydes to the ketone. Overshadowing was found in 6 of the 12 combinations (Table 1), in which after AB training the response to one component was decreased compared to the other [either R_A_(AB) > R_B_(AB) or R_A_(AB) < R_B_(AB), with R_X_(Y) representing the responses to the odour X after training to the odour Y]. All these combinations also fulfilled the second criterion of overshadowing as the response to A or B after mixture training [R_A_(AB) or R_B_(AB)] was lower than the response obtained after training to the single component in the corresponding control group [R_A_(A) or R_B_(B)].

**Figure 3:**
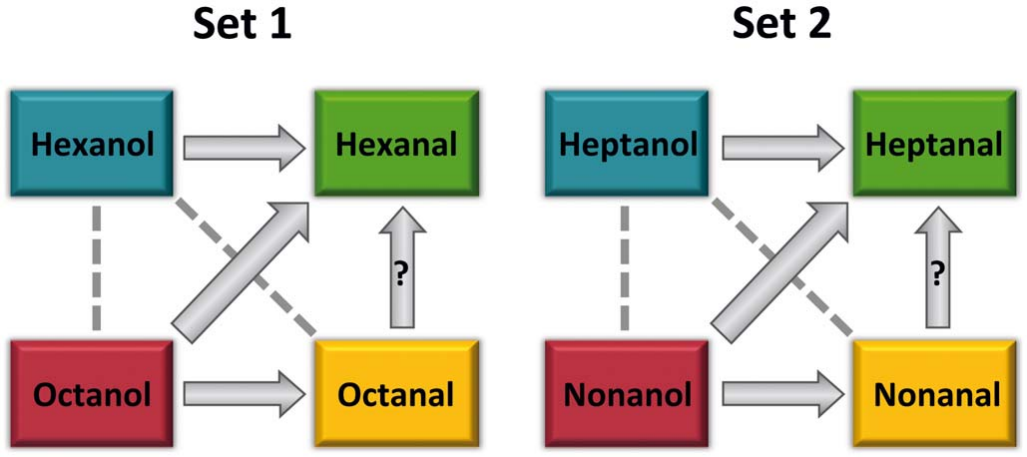
Relationships between odours used in set 1 (left panel) and set 2 (right panel). A solid arrow indicates the occurrence of overshadowing between the components of the mixture. A dotted line indicates the absence of overshadowing. “?” indicates the difficulty in determining the occurrence of overshadowing in this combination of odours due to high levels of generalization from the longer to the shorter aldehyde; the presence of solid arrows linking these odours is suggested by experiment 2. Similar patterns were found between the two sets of odours varying in carbon-chain length, thus indicating that common rules based both on functional group and carbon-chain length determine the occurrence of olfactory overshadowing in ants.

The dominance relationships found in the two sets of odours are summarized in Figure 3. Interestingly, the pattern emerging in both odour sets was similar despite the difference in chain length of the molecules tested. As odorants were equated in their vapour pressure through dilution in mineral oil (Supplementary Table 2), differences in concentration could not account for odour dominance in our experiments, although we cannot exclude possible difference in subjective (perceived) intensity. This result raises therefore the question of whether systematic rules accounting for overshadowing can be uncovered based on the odorant properties that varied in our experiment, namely carbon-chain length and functional group.

### Uncovering the rules underlying overshadowing in olfactory mixtures

Three basic principles account for overshadowing in mixtures composed of two odours. 1) When odour components had the same carbon-chain length but varied in functional group (horizontal relationships in Fig. 3), alcohols always overshadowed aldehydes (e.g. hexanol overshadowed hexanal; Fig. 3, left panel). 2) When odour components had the same functional group but varied in carbon-chain length (vertical relationships in Fig. 3), no overshadowing occurred in the case of alcohols (e.g. no overshadowing between octanol and hexanol; Fig. 3, left panel). Overshadowing determination within aldehydes was difficult to ascertain since only one of the two criteria was fulfilled. 3) When odour components differed not only in carbon-chain length but also in functional group (diagonal relationships in Fig. 3), alcohols with longer carbon-chain length always overshadowed aldehydes with shorter chain length (e.g. octanol overshadowed hexanal; Fig. 3, left panel) whilst no overshadowing occurred between aldehydes with longer carbon-chain length and alcohols with a shorter chain length (e.g. no overshadowing between hexanol and octanal; Fig. 3, left panel).

### The case of aldehydes: is overshadowing masked by generalization?

An interesting finding was the high generalization found between aldehydes. Ants trained with an aldehyde (Supplementary Fig. 5, *Ctrl B* groups; octanal and nonanal) tended to respond not only to the trained odour in the test but also responded highly to the other aldehyde if it had a shorter carbon-chain length (Table 1 and Supplementary Fig. 5, *Ctrl B* groups, hexanal and heptanal). This particular situation raises a problem for the analysis of overshadowing after mixture training. Indeed, in a mixture of two aldehydes differing in carbon-chain length, overshadowing could be masked by the high generalization from the longer to the shorter molecule. For instance, a high generalization from nonanal to heptanal is evident after nonanal training so that responses to both odours did not differ (p_corr_ = 0.22; Table 1 and Supplementary Fig. 5, right, *Ctrl B* group). This result renders the responses of the *OVS* group difficult to interpret: while the responses to nonanal and heptanal (resp. octanal and hexanal) did not differ significantly after training to the mixture of these two odours (Table 1 and Supplementary Fig. 5, *OVS* groups), the response to heptanal (resp. hexanal) was nevertheless significantly lower after mixture training than after heptanal (resp. hexanal) training (Table 1, compare heptanal and hexanal responses of *Ctrl A* and *OVS* groups). Therefore, in the case of aldehydes, only one criterion of overshadowing determination was fulfilled. Was overshadowing masked by the high generalization from longer to shorter aldehydes?

To answer this question we performed a second experiment using three aldehydes varying in carbon-chain length (hexanal, octanal and nonanal), a chemical feature that significantly influences olfactory generalization^30^,^34^. In the previous experiments, aldehydes differed by two carbons in each combination; here we used two other combinations to create a difference in chain length of 1 carbon (octanal/nonanal) and of 3 carbons (hexanal/nonanal). The results obtained with these combinations were compared to those of the heptanal/nonanal combination differing in 2 carbons. We predicted that generalization should be higher in the first combination (octanal/nonanal) due to the higher structural similarity in carbon number, while it should be lower in the second combination (hexanal/nonanal) due to the higher dissimilarity. As a consequence, overshadowing should be visible only in the second case, where it would not be masked by generalization.

**Figure 4:**
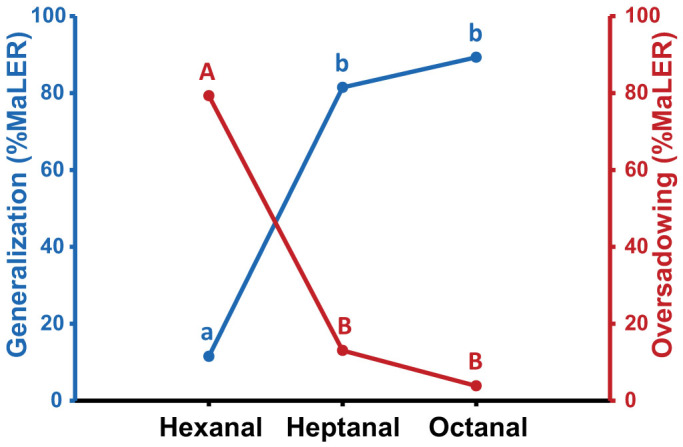
Generalization and overshadowing in aldehydes depending on carbon-chain length. The blue y-axis shows the percentage of ants responding with MaLER to hexanal, heptanal and octanal after nonanal training (i.e. the level of generalization from nonanal to the other aldehydes). The red y-axis represents the difference in the %MaLER of ants responding to nonanal and to the other aldehyde to which it has been combined (hexanal, heptanal or octanal) after mixture training (i.e. the amount of overshadowing). A significant carbon-chain length effect is visible for generalization, which increases when aldehydes are less different in their carbon-chain length (χ^2^ = 27.76; p < 0.001). A significant effect is also visible for the amount of overshadowing, which decreases when aldehydes are less different in their carbon-chain length (χ^2^ = 29.14; p < 0.001). Thus, for the aldehydes tested, the higher the generalization, the lower the amount of overshadowing. Different letters indicate significant differences (sequential Bonferroni corrections after multiple GLMMs).

Indeed, overshadowing was only found in the hexanal/nonanal combination as after mixture training the response to hexanal was lower than the response to nonanal and the response to hexanal was lower after mixture training than that after hexanal training (Table 2). By contrast, none of these criteria was fulfilled after octanal/nonanal training so that no overshadowing was visible. The results obtained for these two odour combinations are shown in Supplementary Fig. 7. As in experiment 1, in both odour combinations the response to the novel odour (a ketone) was low. As predicted, generalization was inversely proportional to the difference in carbon-chain length (Fig. 4, blue curve). It was higher from nonanal to octanal, differing in only 1 carbon (89.29%) while it was lower from nonanal to hexanal, differing in 3 carbons (11.54%). In addition, the difference between the responses to the components after mixture training (amount of overshadowing) increased with component dissimilarity (Fig. 4, red curve; higher in hexanal/nonanal and lower in octanal/nonanal). Therefore, the higher the generalization from nonanal to the other aldehyde, the lower the amount of overshadowing. Taken together, our results suggest that longer aldehydes overshadow shorter aldehydes so that it is very likely that nonanal overshadowed heptanal and that octanal overshadowed hexanal but the effect was masked by generalization (Fig. 3).

## Discussion

In the present study we investigated olfactory overshadowing in ants with the goal of determining how animals perceive and learn complex olfactory blends. In this way, we aimed at providing new insights into the phenomenon of overshadowing, which has been reported in various species on a stimulus-specific basis and without the enouncement of general rules accounting for it. Here, taking into account the two criteria defining olfactory overshadowing, we report a systematic analysis of this phenomenon in ants, for which odour cues and signals play a fundamental role in different aspects of their natural life (e.g., floral odours when foraging, signature mixtures in nestmate recognition)^22^. Our results clearly show that overshadowing critically depends on the combination of at least two parameters investigated in this study: the functional group and the carbon-chain length^34^. Our results thus indicate that olfactory overshadowing obeys specific rules, even in our reduced set of tested odours. Using two comparable sets of odours composed by alcohols and aldehydes that differed or not in carbon-chain length (difference of 0 or 2 carbons), we reveal that (1) overshadowing does not occur in binary mixtures composed by two alcohols; (2) aldehydes with long carbon-chain length overshadow aldehydes with shorter carbon-chain length; (3) alcohols overshadow aldehydes with shorter or equal carbon-chain length.

Our results showed the occurrence of numerous cases of overshadowing among the binary mixtures tested. This shows that this phenomenon is not sporadic^30^ but is widespread in the olfactory perception of ants. Even if we tested only binary mixtures of a reduced set of odour components, this recurrence suggests that, when confronted to a complex olfactory blend, ants would not perceive, learn or retrieve the presence of all blend components but rather guide their choices based on one or few key, dominant components, acting as overshadowing stimuli. As a consequence, the mixture would be reduced to its key components, thereby reducing the necessity of taking into account all the multiple components constituting the olfactory stimulus.

The chemical proprieties of the odours composing the mixture determine their role as either overshadowing or overshadowed component, depending also on the properties of the component with which they are mixed. For instance, octanal overshadowed hexanal but was overshadowed by octanol (Fig. 3). This is in accordance with a recent finding in honeybees showing that a dominant odorant (key odorant) in a given complex mixture of 14 odorants was not necessarily a dominant odorant in another complex mixture^35^.

When binary mixtures are composed of two aldehydes, overshadowing could be masked by the high level of generalization existing between odorants belonging to this functional group. Previous studies have shown that generalization depends on the degree of structural similarity between odours in terms of functional group^30^,^34^,^36^,^37^ and carbon-chain length^34^. High levels of generalization, and a negative correlation between discrimination and the similarity in the number of carbons are well-known phenomena that have been described for aldehydes in bees^34^,^38^, rats^39^ and humans^37^. In ants, decreasing the difference in the number of carbons between aldehydes resulted also in an increase of generalization between odours (Fig. 4). This, in turn, masks overshadowing as no dominant odour could be defined in a binary mixture where both components would be treated equally due to high generalization levels.

Generally, overshadowing is thought to result from differences in stimulus salience^7^,^8^, a factor that can be revealed by differences in component learning^10^. However, in our study, the learning efficiency of the components (acquisition level in the last conditioning trial) did not account for overshadowing as all single odours were equally learnt, and the test responses one hour after conditioning were the same (Fig. 1a, b upper panels). Furthermore, the difference in learning rate did not reflect component dominance. For instance, learning of octanol was slower than that of octanal (see Supplementary Information for detailed analyses of odour learning curves), yet the former overshadowed the latter (Fig. 3). This is in agreement with studies in bees showing that differences in odour learning do not account for olfactory dominance in complex mixtures^35^,^40^.

A recent study showed that *Camponotus aethiops* trained to 15 binary mixtures responded either equally well to both components or more to one component than to the other^30^. Thus, for some mixtures (3 out of the 15 tested) the first criterion for overshadowing was shown. Yet, this study did not compare the response to the dominated component after mixture training to that after component training (i.e. the second criterion of overshadowing). Interestingly, in the three cases revealing a difference in the response to odour components, mixtures were composed by an alcohol and an aldehyde (one case), or by an alcohol and a secondary ketone (two cases). The alcohol was always the dominant odorant and its chain length was either equal (two cases) or longer (one case) than the dominated odorant, which is in agreement with our findings.

In honeybees, training with binary mixtures composed by an aldehyde and an alcohol of equal carbon-chain length (i.e. hexanal + hexanol and octanal + octanol) resulted in the dominance of the aldehyde over the alcohol^14^, contrarily to our results, showing the opposite trend. However, one possible difference between our study and this honeybee study is that the vapour pressure of the odours was not normalized. Odour dominance in a mixture also depends on component concentration, with more concentrated odours dominating less concentrated ones^12^,^35^. Thus, the higher volatility of aldehydes with respect to alcohols having the same carbon-chain length (see Supplementary Table 2) could account for the results of the bee study and explain the discrepancy with our work.

Overshadowing, as detected in our study, could result from interactions both at the peripheral and central levels of odour processing. At the peripheral level, overshadowing could result from the competition of mixture components for olfactory receptor (OR) binding sites^41^,^42^,^43^. Indeed, most ORs are broadly tuned to specific olfactory ligands and are sensitive to structural similarity^44^, thus syntopic interactions (either agonist or antagonist competitions at the OR binding site)^45^,^46^ could account for overshadowing. At the central level, olfactory stimuli are first processed in the antennal lobes (ALs), the primary olfactory neuropil in the insect brain. In ants, each AL consists of several hundreds of glomeruli (around 450 glomeruli have been identified in worker carpenter ants^31^,^47^,^48^,^49^). Glomeruli are functional units receiving input from olfactory receptor neurons (ORNs) and conveying the information to higher-order structures such as the mushroom bodies (MB) via projection neurons (PNs). Glomeruli interact through local interneurons (LNs) that are mostly inhibitory. Imaging studies have shown that the glomerular activity pattern of binary mixtures at the AL input level is similar to the combination of the patterns evoked by the mixture components^50^,^51^,^52^,^53^,^54^. Interestingly, in the honeybee, the mixture activity pattern is always more similar to that of the stronger component (i.e. the one which activated the higher number of glomeruli)^51^. Thus, overshadowing could result from the fact that the overshadowing component activates a higher number of glomeruli in the AL than the overshadowed one. At the output of the AL, mixture representation is less similar to the mere sum of its components^53^,^54^,^55^. This indicates that olfactory processing within the AL network increases the uniqueness (holistic processing) of the mixture. Mixture interactions at the AL input and output levels are thought to result from the action of LNs. These interneurons are thus good candidates as mediators or facilitators of overshadowing. By reducing the level of activation of some glomeruli, LNs could sharp the mixture representation so that this representation becomes more similar to representation of one component (the overshadowing one) than to that of the other component (the overshadowed one). Furthermore, feedbacks from MB to AL^56^ could also modulate the mixture representation in the AL.

We hypothesize that in the cases where overshadowing occurs, the representation of the binary mixture in the AL should be more similar to the overshadowing component than to the overshadowed one; whereas in the cases of absence of overshadowing the representation of the binary mixture in the AL should be more similar to the combination of both components. This hypothesis is based on findings in rats^39^ and bees^34^ showing that perceptual similarity of single odours can be predicted from the similarities in the neural response evoked by these odours in the primary olfactory centre: the AL in insects and the olfactory bulb in vertebrates. Thus, the high generalization level between the binary mixture and the overshadowing component and the low generalization level from the overshadowed component to the mixture should translate in terms of similarity in glomerular activity pattern. Since olfactory learning modifies odour representation in the AL^57^,^58^,^59^, it is conceivable that the neural representation of the mixture becomes more similar to that of the overshadowing component along successive trials.

Moreover, it is possible that overshadowing does not occur exclusively at the level of AL processing but involves also higher order brain structures such as the MB or the lateral horn. Odorant salience could be modulated at these levels, therefore determining odour-dominance relationships beyond the olfactory coding occurring in the AL. Further studies on olfactory mixture processing using electrophysiological and optophysiological recording techniques recently developed in ants^23^,^31^,^32^,^60^,^61^,^62^,^63^ will contribute to our understanding of how olfactory mixtures are perceived by insects, and whether overshadowing results from interactions at the peripheral and/or central levels and/or from a learning-induced variation in odour representation in the AL.

## Methods

### Study organism

Seventeen colonies of *Camponotus aethiops* were collected in May 2012 and April 2013 at Pompertuzat (Midi-Pyrénées, France, latitude 43.5°, longitude 1.516667°) and kept in the laboratory (25°C, light-dark cycle = 12:12, 70% humidity) in artificial nests composed of two plastic boxes connected by a plastic hose. One of the boxes was covered by cardboard and contained a plaster floor to form the nest; the other box, exposed to light constituted the foraging arena. The inner sides of the two boxes were coated with Fluon® to prevent ants from escaping. Ants were fed twice a week with carbohydrates, protein and vitamins^64^; water was provided *ad libitum*. Three weeks before the onset of the experiment, the ants were deprived of sucrose to increase their appetitive motivation for sucrose reward during conditioning; mealworms (protein) and water were still provided *ad libitum*.

### Handling

Medium sized workers collected in the foraging arena were kept in glass vials and cooled on ice during 10 min until they stopped moving. Each ant was then harnessed in an individual holder made of a 0.2 ml Eppendorf® tube from which the tip was removed. The head of the ant was passed through the apical hole and a strip of adhesive tape was placed between the head and the thorax to prevent body movements (Supplementary Fig. 8). The antennae and mouthparts could, nevertheless, freely move^26^. Ants were then left for 3 h to recover from anaesthesia and habituate to harnessing conditions.

### Stimuli

In the first experiment, two sets of four odours (set 1: hexanol, hexanal, octanol, octanal; set 2: heptanol, heptanal, nonanol, nonanal, Sigma Aldrich, France) and their binary mixtures (6 binary mixtures by set of odours, 12 in total) were used as conditioning stimuli (CS). Odours differed in their functional group (alcohols and aldehydes) and chain length (set 1: 6 vs. 8 carbons; set 2: 7 vs. 9 carbons).

In the second experiment, three aldehydes (hexanal, octanal and nonanal) and two binary mixtures (hexanal + nonanal and octanal + nonanal), which were not tested in experiment 1, were used as conditioning stimuli (CS). Odours differed only in their carbon-chain length (6, 8 and 9 carbons).

In both experiments, an additional odour (C) was used in the test performed after conditioning in order to measure the tendency to generalise olfactory responses to a novel odour. The four novel odours used differed from the CS in their functional group (ketones) (see Supplementary Table 1).

Vapour pressures of all odours were standardized by diluting the pure substances in mineral oil (see Supplementary Table 2). Six microliters of single odour or binary mixture were applied onto a 1 cm^2^ piece of filter paper, which was inserted into a plastic 10 ml syringe used to stimulate the ants. The unconditioned stimulus (US) was 50% sucrose solution (w/w).

### Experimental design

Ants were subjected to Pavlovian conditioning of the *Maxilla-Labium* extension response (MaLER)^26^. Three groups of ants were conditioned in parallel for each combination of two odours A and B: one group was conditioned to the binary mixture AB (*OVS* group) while the other two control groups were conditioned to the odour components (A and B for *Ctrl A* and *Ctrl B* groups, respectively). Conditioning consisted in six consecutive trials of paired CS-US presentation. Each trial lasted 1 min. Twenty-five seconds after placing the ant under a binocular, the CS was presented at 2 cm of the antennae by blowing an air puff during 5 s; a sucrose droplet eliciting the MaLER was presented during 5 s during which individuals were allowed to drink the solution. Sucrose delivery started 3 s after odour onset thus defining a CS-US overlap of 2 s. The ant was then left during 27 s in the conditioning set-up in order to avoid context learning. Because 15 ants were trained in a row, the inter-trial interval (ITI) was 15 min. An air extractor was placed behind the ant in order to remove undesired odour stimuli. Individuals that did not respond at least three times to the US were discarded (1.46% of the tested individuals).

One hour after training, the three groups (*OVS* group, *Ctrl A* and *Ctrl B* group) were tested with the binary mixture AB, each of the odour components A and B and a novel odour C in the absence of reward. Each odour was presented during 5 s and the sequence of odour presentation was random. Similarly to training trials, each test lasted 1 min, resulting in an ITI of 15 min. Mortality rate during the experiments was of 2.76%.

### Data analysis

All statistical analyses were performed with R-2.15.0. Upon odour presentation, the response of the ants was scored as 1 when MaLER was visible and 0 otherwise. The percentage of conditioned responses was then calculated.

Generalized linear mixed models (GLMM, package lme4, Bates et al. 2011. *lme4: Linear mixed-effects models using S4 classes*. R package version 0.999999-0) with a binomial error structure (logit-link) were used to analyse learning efficiency, measured in terms of the % of conditioned responses reached in the last conditioning trial in the case of single substances and binary mixtures of set 1 and set 2. The ant's response was used as response variable, the colony of origin was included as random factor in order to adjust for colony origin, and the last trial constituted the subset of data.

Comparisons between single odours and binary mixtures within each set were tested by including the odour composition (single and binary) as fixed factor, the colony of origin as random factors and the odour set as subset of data. A single odour has been conditioned in three different odour combinations (e.g. hexanol has been conditioned when studying overshadowing in odour combinations hexanol/hexanal, hexanol/octanol and hexanol/octanal). Because the three different odour combinations containing the same single odour could have been studied at different moments of the year, we included the odour combination as random factor to adjust for a potential seasonal effect on the colonies' state and thus on learning abilities.

Differences between set 1 and set 2 for single odours and binary mixtures were revealed by coding the set as fixed factor, the odour combination (for single substances) and the colony of origin as random factor and the odour composition (single or binary) as subset of data.

Differences in the ants' response to the conditioned odour (single odours or binary mixture) between the last trial of the conditioning and the test were detected by means of a McNemar's Chi Square test.

The responses of each group (*OVS* group, *Ctrl A* and *Ctrl B*) to the four odours tested (A, B, AB and C) one hour after conditioning, were compared with Cochran's Q tests. Multiple McNemar's Chi Square tests (with sequential Bonferroni corrections) were applied for pairwise comparisons between test odours. For instance for the *OVS* group, these tests were applied to compare the responses to A, B, AB and C after mixture training. A difference in the response to A and B of this group (that is a difference between R_A_(AB) and R_B_(AB), see Table 1) was used as a criterion for overshadowing. Furthermore, we used Fisher's Exact Test to compare the response to the mixture components of the *OVS* group [e.g. R_A_(AB) ] with that of the control group trained to the component considered [e.g. R_A_(A)] (see Table 1). This additional comparison constitutes the second criterion for determining the occurrence of overshadowing.

In experiment 2, to ensure that in the test we analysed a true generalization response from nonanal to the other aldehydes (hexanal, heptanal or octanal), we only kept ants that successfully learnt nonanal (*Ctrl B* group) in each odour combination (96.30; 93.10 and 96.55% of ants respectively for odour combination hexanal/nonanal, heptanal/nonanal and octanal/nonanal). To test whether the level of generalization from nonanal to the other aldehyde was dependent on the carbon-chain length of the other aldehyde (6, 7 and 8 respectively) we used a GLMM with a binomial error structure (logit-link). The ant's response to the other aldehyde was used as response variable; the carbon-chain length of the other aldehyde was included as fixed factor and the colony of origin as random factor. Post-hoc differences were revealed by applying the same GLMMs to the respectively reduced set of data and adjusting the p-values with sequential Bonferroni corrections.

As for generalization, to ensure that in the test we analysed a true “overshadowing response”, we only kept ants that had successfully learnt the mixture of nonanal and the other aldehyde (*OVS* group; 100; 82.14 and 92.86% respectively for odour combination hexanal/nonanal, heptanal/nonanal and octanal/nonanal). For each ant, we calculated an “overshadowing response” by subtracting its response to the other aldehyde from its response to nonanal. As no ant of the *OVS* group responded to the other aldehyde without responding to nonanal, the “overshadowing response” was 0 (ants that did not respond to any of the components or that responded to both components) or 1 (ants that responded only to nonanal).

A GLMM with a binomial error structure (logit-link) was used to test whether the amount of overshadowing between nonanal and the other aldehyde depended on the carbon-chain length of the aldehyde mixed with nonanal. The “overshadowing response” (see above) was used as response variable; the carbon-chain length of the other aldehyde was included as fixed factor and the colony of origin as random factor. Post-hoc differences were revealed by applying the same GLMMs to the respectively reduced set of data and adjusting the p-values with sequential Bonferroni corrections.

## Author Contributions

M.G., P.d.E. and M.P. designed research; M.P. performed research and analysed data; M.P., M.G. and P.d.E. wrote the paper.

## Supplementary Material

Supplementary Information Supplementary Information

## Figures and Tables

**Table 1:** Test responses and overshadowing criteria for odour combinations used in set 1 and set 2

		*Ctrl A*			*Ctrl B*			*OVS*							
	Combination of odours (A/B)	n	R_A_(A)	R_B_(A)	n	R_B_(B)	R_A_(B)	n	R_AB_(AB)	R_A_(AB)	R_B_(AB)	R_A_(AB) ≠ R_B_(AB) (p-value)	R_A_(A) ≠ R_A_(AB) (p-value)	R_B_(B) ≠ R_B_(AB) (p-value)	Overshadowing
**Set 1**	**hexanol/hexanal**	29	96.55	27.59	27	96.30	11.11	28	92.86	64.71	47.06	**<0.001**	0.35	**<0.001**	**A > B**
	**hexanol/octanol**	26	84.62	23.08	25	84.00	28.00	29	82.76	62.07	82.76	0.23	0.08	1	A = B
	**hexanol/octanal**	31	77.42	6.45	31	80.65	9.68	34	73.53	47.06	64.71	0.15	**<0.05**	0.18	A = B
	**hexanal/octanol**	29	82.76	0.00	29	96.55	3.45	29	93.10	6.90	93.10	**<0.001**	**<0.001**	1	**B > A**
	**hexanal/octanal**	30	90.00	50.00	29	89.66	58.62	28	92.86	60.71	89.29	0.054 (t)	**<0.05**	1	A = B (?)
	**octanol/octanal**	28	85.71	0.00	29	89.66	6.90	29	96.55	93.10	3.45	**<0.001**	0.42	**<0.001**	**A > B**
**Set 2**	**heptanol/heptanal**	29	93.10	0.00	29	93.10	24.14	29	93.10	96.55	3.45	**<0.001**	1	**<0.001**	**A > B**
	**heptanol/nonanol**	29	72.41	17.24	28	78.57	35.17	28	85.71	67.86	78.57	0.50	0.78	1	A = B
	**heptanol/nonanal**	32	84.38	3.13	34	88.24	5.88	34	85.29	79.41	82.35	1	0.75	0.73	A = B
	**heptanal/nonanol**	33	63.64	3.03	32	81.25	3.13	32	87.50	0.00	68.75	**<0.001**	**<0.001**	0.39	**B > A**
	**heptanal/nonanal**	29	89.66	27.59	29	93.10	75.86	28	82.14	64.29	78.57	0.27	**<0.05**	0.14	A = B (?)
	**nonanol/nonanal**	28	92.86	7.14	29	79.31	13.79	28	85.71	71.43	32.14	**<0.05**	0.08	**<0.001**	**A > B**

The table shows, for each odour combination, the sample size (n) and the responses of the control groups (*Ctrl A* and *Ctrl B*) and of the group trained with the binary mixture (*OVS*) in the test performed 1 h after training. Values of R_X_(Y) represent the percentage of ants responding with MaLER to the odour X after training to the odour Y. A difference in responses to the mixture components of the *OVS* group [i.e. R_A_(AB) and R_B_(AB)], first criterion for overshadowing determination, is indicated by adjusted p-values of pairwise response comparisons (sequential Bonferroni corrections after multiple McNemar's chi square tests). Fishers exact tests were used to compare responses to the mixture components of the *OVS* group with responses of the control groups to their training odour [i.e. R_A_(A) with R_A_(AB) and R_B_(B) with R_B_(AB)] to assess the second criterion for overshadowing determination. Significant differences are indicated in bold. “X = Y”: no overshadowing; “X > Y”: X overshadowed Y (where X and Y are either A and B or B and A) as shown by the lower response to Y after mixture training and by the reduction of responses to Y after mixture training compared to responses to Y after Y training. “?” indicates difficulty in determining the occurrence of overshadowing due to high levels of generalization from the longer to the shorter aldehyde (further investigated in experiment 2). “t” indicates tendency (p approaching significance).

**Table 2:** Test responses and overshadowing criteria for aldehyde combinations used in experiment 2

	*Ctrl A*		*Ctrl B*		*OVS*						
Combination of odours (A/B)	R_A_(A)	R_B_(A)	R_B_(B)	R_A_(B)	R_AB_(AB)	R_A_(AB)	R_B_(AB)	R_A_(AB) ≠ R_B_(AB) (p-value)	R_A_(A) ≠ R_A_(AB) (p-value)	R_B_(B) ≠ R_B_(AB) (p-value)	Overshadowing
**hexanal/nonanal**	93.10	13.79	96.30	11.11	100.00	17.24	96.55	**<0.001**	**<0.001**	1	B > A
**heptanal/nonanal**	89.66	27.59	93.10	75.86	82.14	64.29	78.57	0.27	**<0.05**	0.14	A = B (?)
**octanal/nonanal**	85.71	67.86	96.55	86.21	92.86	96.43	100.00	1.00	0.35	1	A = B (?)

The table shows, for each odour combination, the sample size (n) and the responses of the control groups (*Ctrl A* and *Ctrl B*) and of the group trained with the mixture (*OVS*) in the test performed 1 h after training. Values of R_X_(Y) represent the percentage of ants responding with MaLER to the odour X after training to the odour Y. A difference in responses to the mixture components of the *OVS* group [i.e. R_A_(AB) and R_B_(AB)], first criterion for overshadowing determination, is indicated by adjusted p-values of pairwise response comparisons (sequential Bonferroni corrections after multiple McNemar's chi square tests). Fishers exact tests were used to compare responses to the mixture components of the *OVS* group with responses of the control groups to their training odour [i.e. R_A_(A) with R_A_(AB) and R_B_(B) with R_B_(AB)] to assess the second criterion for overshadowing determination. Response levels and statistical differences of the heptanal/nonanal combination are extracted from Experiment 1 (see Table 1). Significant differences are indicated in bold. “X = Y”: no overshadowing; “X > Y”: X overshadowed Y (where X and Y are either A and B or B and A) as shown by the lower response to Y after mixture training and by the reduction of responses to Y after mixture training compared to responses to Y after Y training. “?” indicates difficulty in determining the occurrence of overshadowing due to high levels of generalization from nonanal to heptanal and from nonanal to octanal.
