# Supplementary material for: The scent of mixtures: rules of odour processing in ants
Source: Sci Rep. 2015 Mar 2;5:8659. doi: 10.1038/srep08659 (PMC4345350; doi:10.1038/srep08659)
Supplement: Supplementary Information [file srep08659-s1.pdf]

Supplementary Information

The scent of mixtures: rules of odour processing in ants

Margot Perez, Martin Giurfa and Patrizia d'Ettorre

Statistical analyses for learning curves of single odours and binary mixtures

Differences in acquisition between single odours or between binary mixtures within each odour set were tested using generalized linear mixed models (GLMM, package lme4[1]) with a binomial error structure (logit-link). The ant's *maxilla-labium* extension response (MaLER)[2], scored 0 or 1,was used as response variable. Trials were used as predictor variable (covariate), the odour as fixed factor and individuals and colony of origin as random factors in order to account for repeated measurements and adjust for colony origin. Each single odour was conditioned in three different odour combinations (e.g. hexanol has been conditioned in combinations hexanol/hexanal, hexanol/octanol and hexanol/octanal). Because the three combinations containing the same single odour were in some cases tested at different times of the year, we included odour combination as random factor to adjust for a potential seasonal effect on the colonies' state and on learning abilities. Post-hoc differences between single odours or binary mixtures within each set were revealed by applying the same GLMMs to the respectively reduced set of data, and adjusting the p-value with sequential Bonferroni correction.

Ants efficiently learnt the odour-sucrose association, both in the case of single odours and binary mixtures, and exhibited a high level (approximately 85%) of conditioned responses in the last training trial (see main text, Fig. 1). However, we found a significant interaction between trials and

odour in the case of single substances in set 2 (χ^2=7.83; p<0.05), indicating that acquisition rates varied among single odours. We also observed a significant effect of odour in set 1 (single odours: χ^2=18.87; p<0.001; binary mixtures: χ^2=11.26; p<0.05) and for single odours in set 2 (χ^2=14.30; p<0.01), revealing that some odours are better learnt than others. Post-hoc tests showed that (1) hexanol and octanal were better learnt than octanol (Fig. 1a, upper panel) and (2) the hexanol + hexanal mixture was better learnt than the hexanol + octanol mixture (Fig. 1a, lower panel). In the case of single substances of set 2, we did not find significant post-hoc differences between odours after correction of the alpha-level; yet, nonanal tended to be better learnt than heptanol (p_{corr}=0.061).

References

1. Bates, D., Maechler, M. & Bolker, B. *lme4: Linear mixed-effects models using S4 classes*. (2011). at <http://lme4.r-forge.r-project.org/>
2. Guerrieri, F. J. & d'Ettorre, P. Associative learning in ants: conditioning of the maxilla-labium extension response in *Camponotus aethiops*. *J. Insect Physiol.* **56,** 88–92 (2010).

Supplementary Table 1

	Combination of odours	Novel odour
**Set 1**	hexanol/hexanal	2-nonanone
	hexanol/octanol	2-nonanone
	hexanol/octanal	2-hexanone
	hexanal/octanol	2-heptanone
	hexanal/octanal	2-nonanone
	octanol/octanal	2-nonanone
**Set 2**	heptanol/heptanal	2-nonanone
	heptanol/nonanol	2-octanone
	heptanol/nonanal	2-octanone
	heptanal/nonanol	2-octanone
	heptanal/nonanal	2-octanone
	nonanol/nonanal	2-octanone

Supplementary Table 1: Odour combinations of alcohols and aldehydes presented in sets 1 and 2 with the ketone used as a novel odour in the tests. The ketone tested was unknown to the ants and allowed to measure the tendency to generalise olfactory responses.

Supplementary Table 2

	Odours	Purity (%)	VP [mmHg] 25°C	Dilution (in 1 ml)	
				µl odour (1/VP*10)	µl mineral oil
Set 1	hexanol	99	0,93	10,8	989,2
	hexanal	97	11,3	0,9	999,1
	octanol	99,5	0,08	125	875
	octanal	99	1,18	8,5	991,5
Set 2	heptanol	99	0,22	45,5	954,5
	heptanal	95	3,52	2,8	997,2
	nonanol	98	0,02	500	500
	nonanal	95	0,37	27	973
Novel	2-hexanone	98	11,6	0,9	999,1
	2-heptanone	98	3,85	2,6	997,4
	2-octanone	98	1,35	7,4	992,6
	2-nonanone	99	0,65	15,4	984,6

Supplementary Table 2: Chemical characteristics of the odours used in sets 1 and 2, and of the novel test odours. Purity (commercial description of the product), vapour pressure (VP) and dilution quantities in mineral oil are indicated for each odour.

Supplementary Figure 1

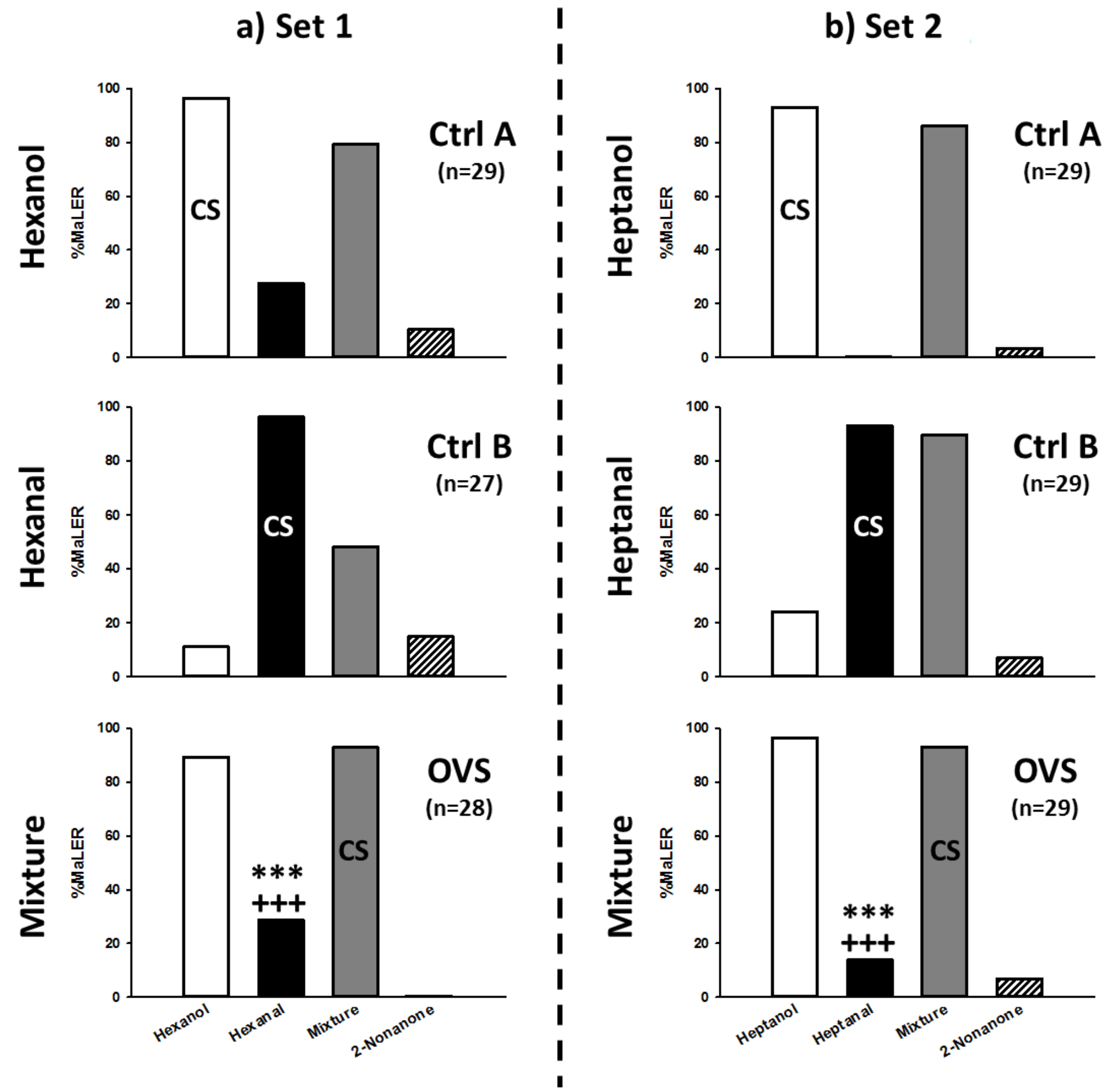

Supplementary Figure 1: **Test responses to combinations of an alcohol and an aldehyde with the same and the shortest carbon-chain length of each odour set (set 1: hexanol/hexanal; set 2: heptanol/heptanal). a)** Responses of the control groups (*Ctrl A*: hexanol; *Ctrl B*: hexanal) and the group trained to the mixture (*OVS*: hexanol + hexanal). White bars represent responses to hexanol, black bars responses to hexanal, grey bars responses to the mixture and hatched bars responses to the novel odour tested for generalization (2-nonanone). **b)** Responses of the control groups (*Ctrl A*: heptanol; *Ctrl B*: heptanal) and the group trained to the mixture (*OVS*: heptanol + heptanal). White bars represent responses to heptanol, black bars responses to heptanal, grey bars responses to the mixture and hatched bars responses to the novel odour tested for generalization (2-nonanone). Sample size (n) of each group for both odour sets is indicated in parentheses. Overshadowing occurred in both mixtures as shown by the lower responses to one odour after mixture training (white vs. black bar in *OVS* groups: ***; $p < 0.001$) and by the reduction of responses to that odour after mixture training compared to responses after training to the odour alone (comparison of black bars of *Ctrl B* and *OVS* groups: +++; $p < 0.001$).

Supplementary Figure 2

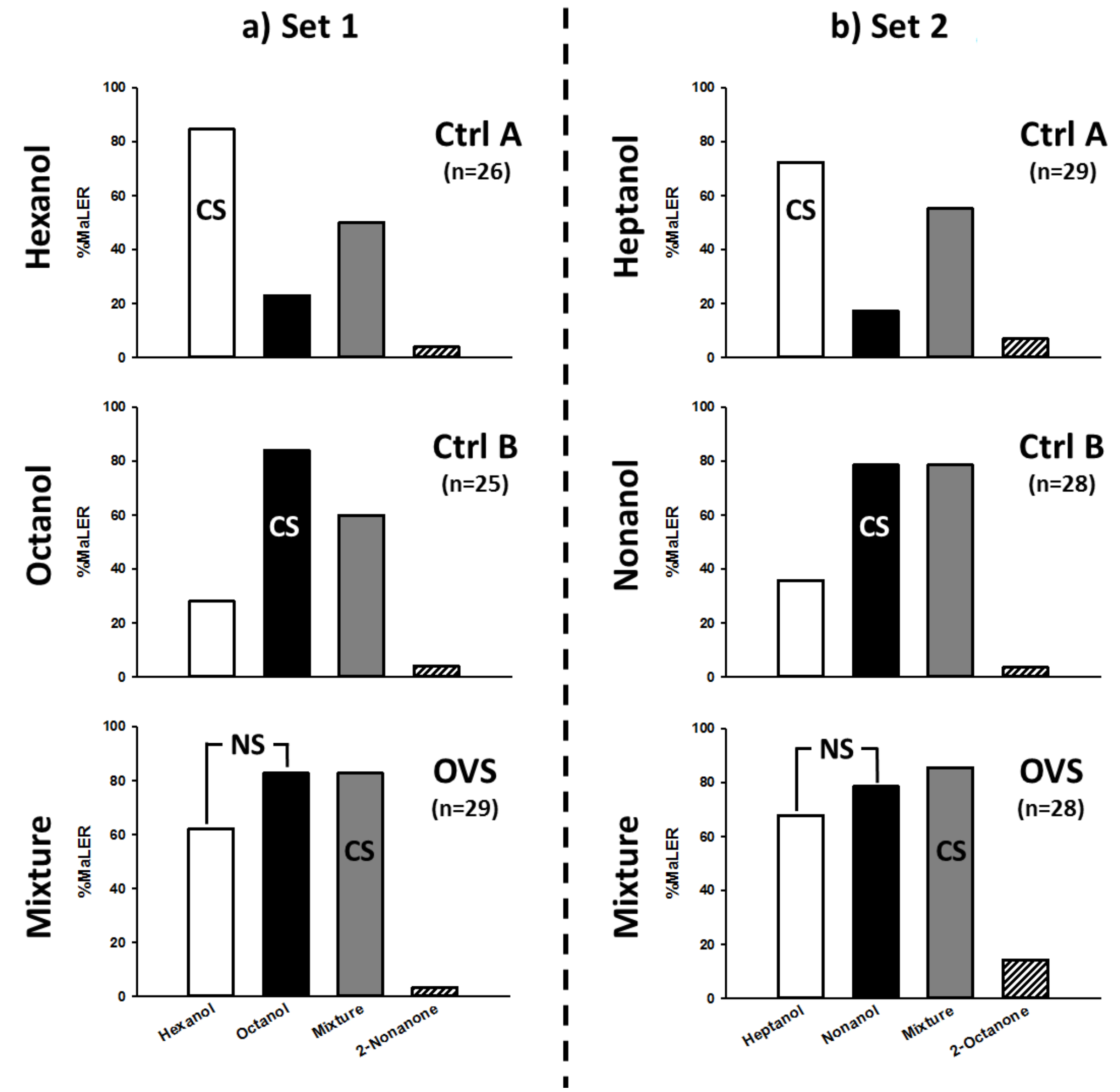

Supplementary Figure 2: **Test responses to combinations of two alcohols with carbon-chain lengths differing in two carbons in odour sets 1 (hexanol/octanol) and set 2 (heptanol/nonanol). a)** Responses of the control groups (*Ctrl A*: hexanol; *Ctrl B*: octanol) and the group trained to the mixture (*OVS*: hexanol + octanol). White bars represent responses to hexanol, black bars responses to octanol, grey bars responses to the mixture and hatched bars responses to the novel odour tested for generalization (2-nonanone). **b)** Responses of the control groups (*Ctrl A*: heptanol; *Ctrl B*: nonanol) and the group trained to the mixture (*OVS*: heptanol + nonanol). White bars represent responses to heptanol, black bars responses to nonanol, grey bars responses to the mixture and hatched bars responses to the novel odour tested for generalization (2-octanone). Sample size (n) of each group for both odour sets is indicated in parentheses. No overshadowing occurred in both mixtures as shown by similar high levels of responses to both components after mixture training (white vs. black bar in *OVS* groups: NS, non-significant).

Supplementary Figure 3

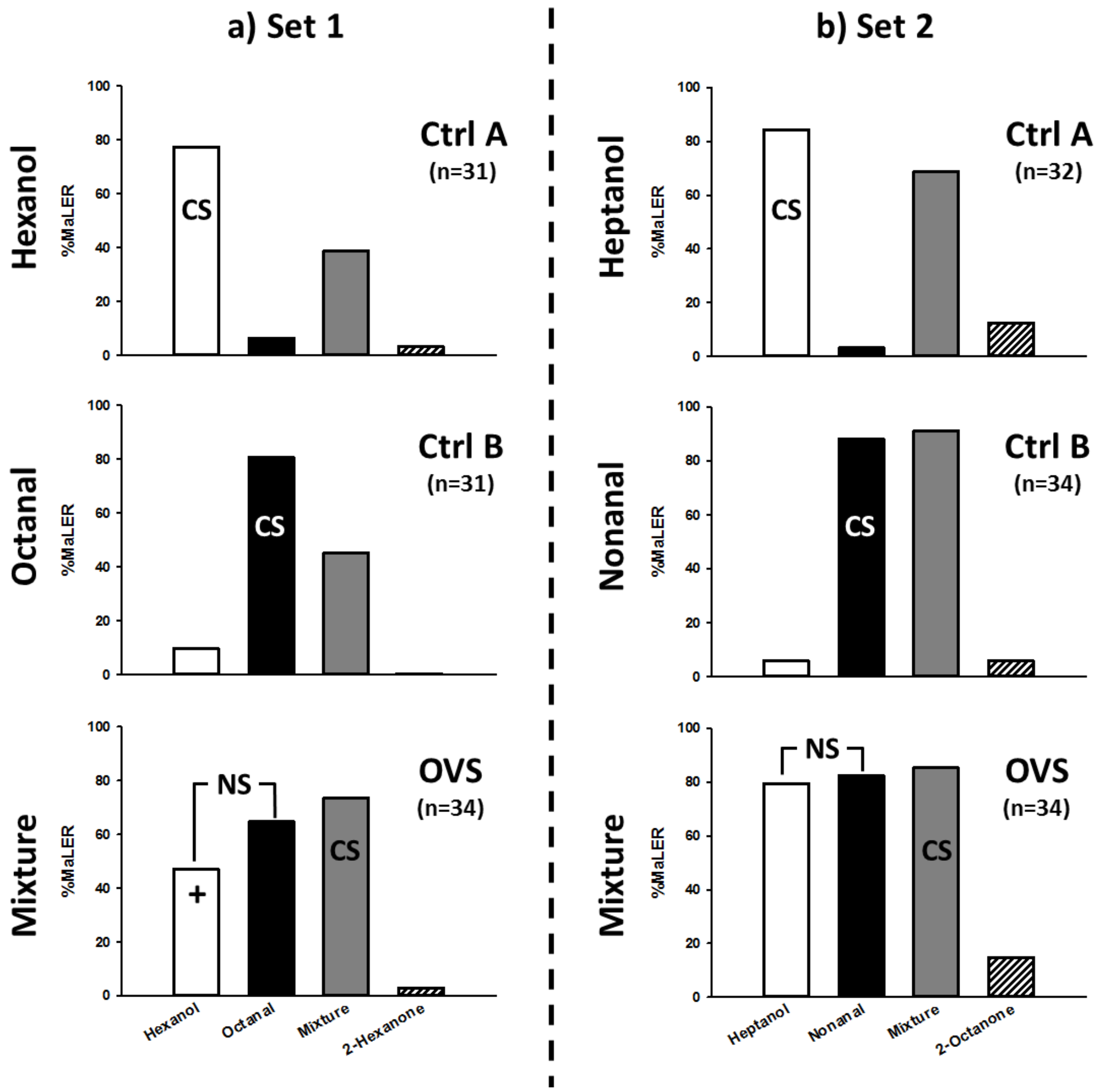

Supplementary Figure 3: **Test responses to combinations of an alcohol with the shortest carbon-chain length and an aldehyde with the longest carbon-chain length of each odour set (set1: hexanol/octanal; set2: heptanol/nonanal). a)** Responses of the control groups (*Ctrl A*: hexanol; *Ctrl B*: octanal) and the group trained to the mixture (*OVS*: hexanol + octanal). White bars represent responses to hexanol, black bars responses to octanal, grey bars responses to the mixture and hatched bars responses to the novel odour tested for generalization (2-hexanone). **b)** Responses of the control groups (*Ctrl A*: heptanol; *Ctrl B*: nonanal) and the group trained to the mixture (*OVS*: heptanol + nonanal). White bars represent responses to heptanol, black bars responses to nonanal, grey bars responses to the mixture and hatched bars responses to the novel odour tested for generalization (2-octanone). Sample size (n) of each group for both odour sets is indicated in parentheses. No overshadowing occurred in both mixtures as shown by similar high levels of responses to both components after mixture training (white vs. black bar in *OVS* groups: NS, non-significant), although responses to hexanol were significantly lower after mixture training than responses after training to hexanol alone (+; $p<0.05$).

Supplementary Figure 4

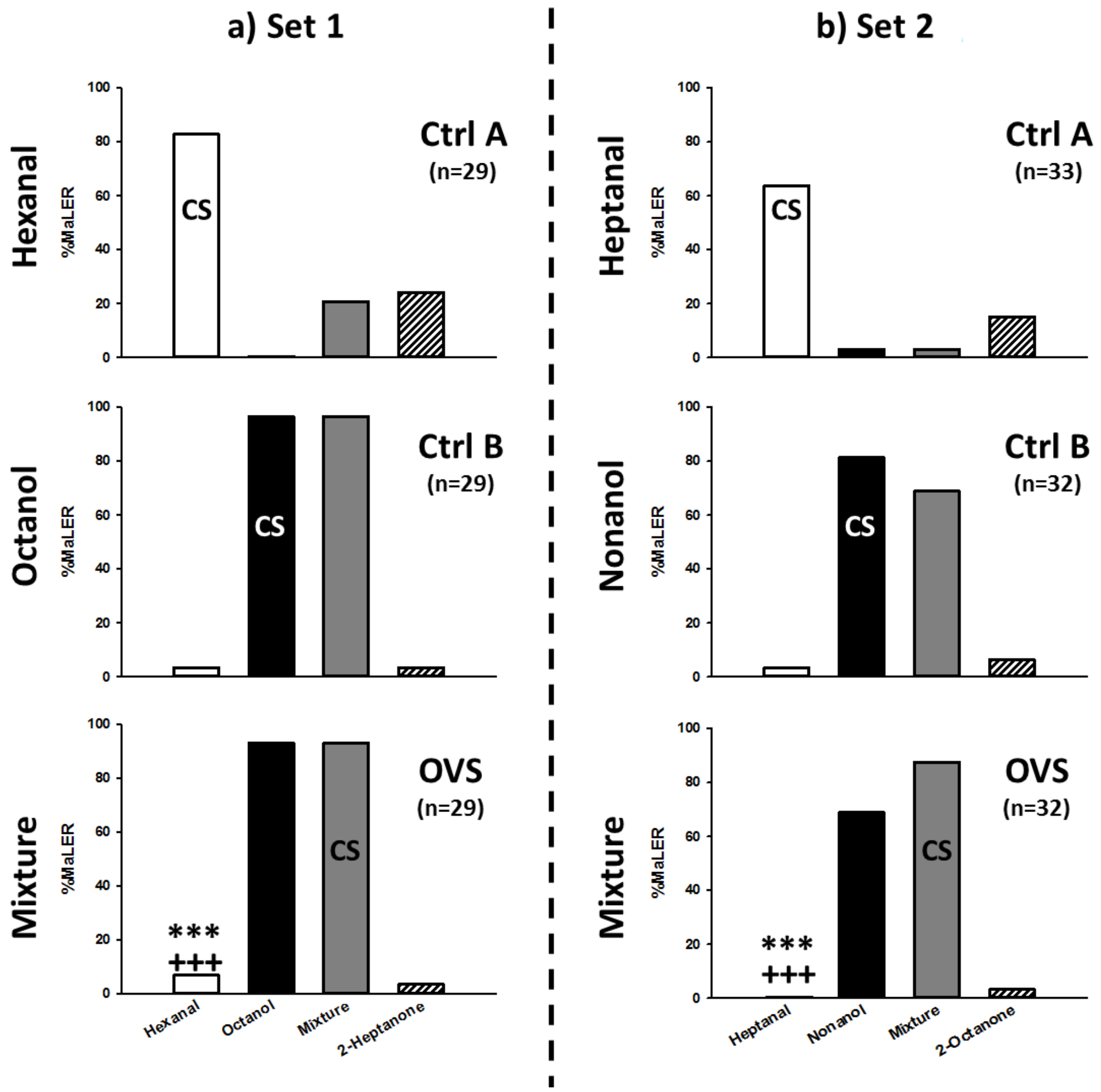

Supplementary Figure 4: **Test responses to combinations of an alcohol with the longest carbon-chain length and an aldehyde with the shortest carbon-chain length of each odour set (set 1: hexanal/octanol; set 2: heptanal/nonanol). a)** Responses of the control groups (*Ctrl A*: hexanal; *Ctrl B*: octanol) and the group trained to the mixture (*OVS*: hexanal + octanol). White bars represent responses to hexanal, black bars responses to octanol, grey bars responses to the mixture and hatched bars responses to the novel odour tested for generalization (2-heptanone). **b)** Responses of the control groups (*Ctrl A*: heptanal; *Ctrl B*: nonanol) and the group trained to the mixture (*OVS*: heptanal + nonanol). White bars represent responses to heptanal, black bars responses to nonanol, grey bars responses to the mixture and hatched bars responses to the novel odour tested for generalization (2-octanone). Sample size (n) of each group for both odour sets is indicated in parentheses. Overshadowing occurred in both mixtures as shown by the lower responses to one odour after mixture training (white vs. black bar in *OVS* groups: ***; $p < 0.001$) and by the reduction of responses to that odour after mixture training compared to responses after training to the odour alone (comparison of white bars of *Ctrl A* and *OVS* groups: +++; $p < 0.001$).

Supplementary Figure 5

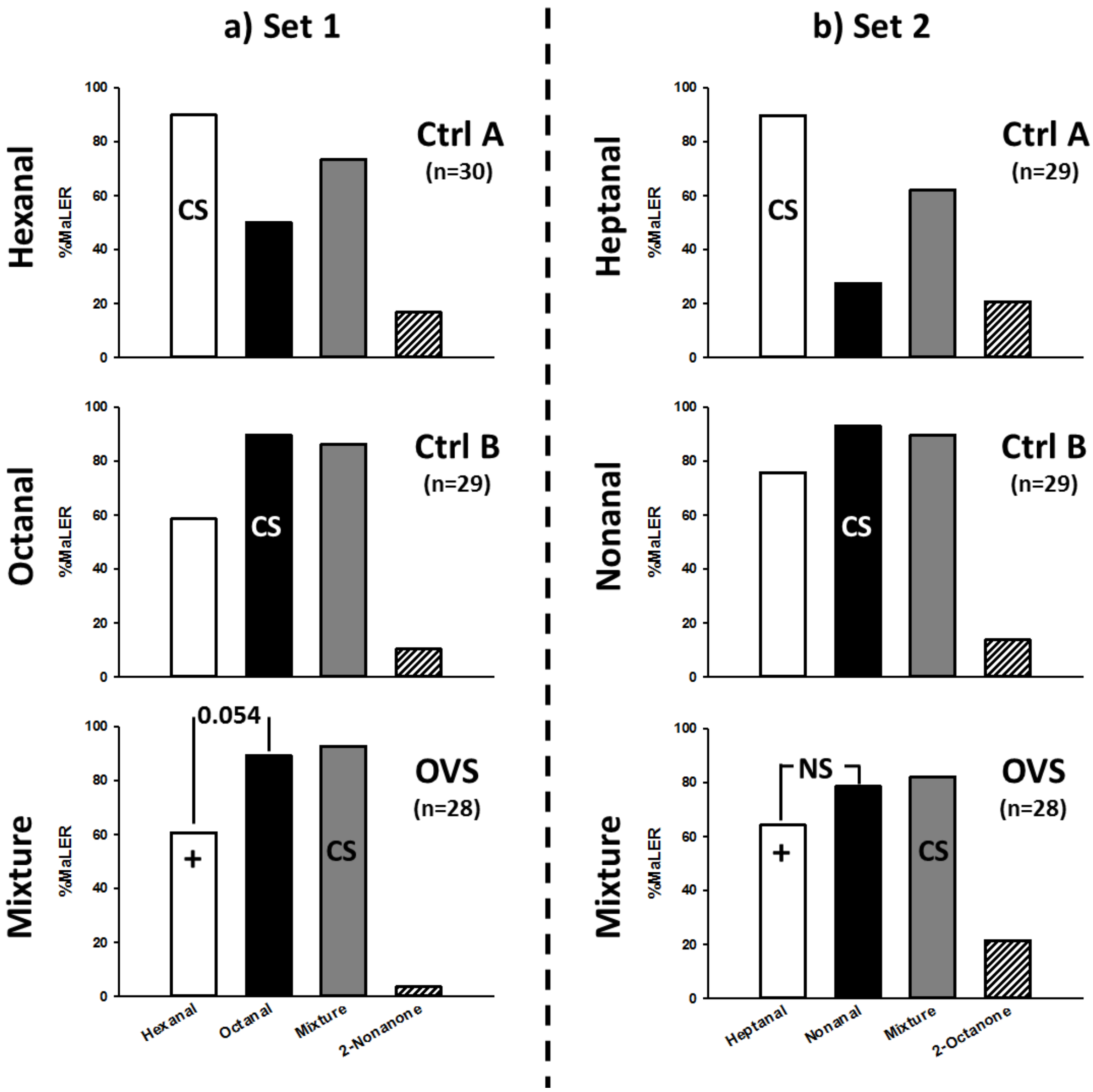

Supplementary Figure 5: **Test responses to combinations of two aldehydes with carbon-chain lengths differing in two carbons in odour sets 1 (hexanal/octanal) and set 2 (heptanal/nonanal). a)** Responses of the control groups (*Ctrl A*: hexanal; *Ctrl B*: octanal) and the group trained to the mixture (*OVS*: hexanal + octanal). White bars represent responses to hexanal, black bars responses to octanal, grey bars responses to the mixture and hatched bars responses to the novel odour tested for generalization (2-nonanone). **b)** Responses of the control groups (*Ctrl A*: heptanal; *Ctrl B*: nonanal) and the group trained to the mixture (*OVS*: heptanal + nonanal). White bars represent responses to heptanal, black bars responses to nonanal, grey bars responses to the mixture and hatched bars responses to the novel odour tested for generalization (2-octanone). Sample size (n) of each group for both odour sets is indicated in parentheses. The high level of generalization from the longer to the shorter aldehyde (white vs. black bars within *Ctrl B* groups) determined that responses to these odours were similarly high after mixture training (white and black bars of the *OVS* groups). Thus, no overshadowing effect could be detected in these cases.

Supplementary Figure 6

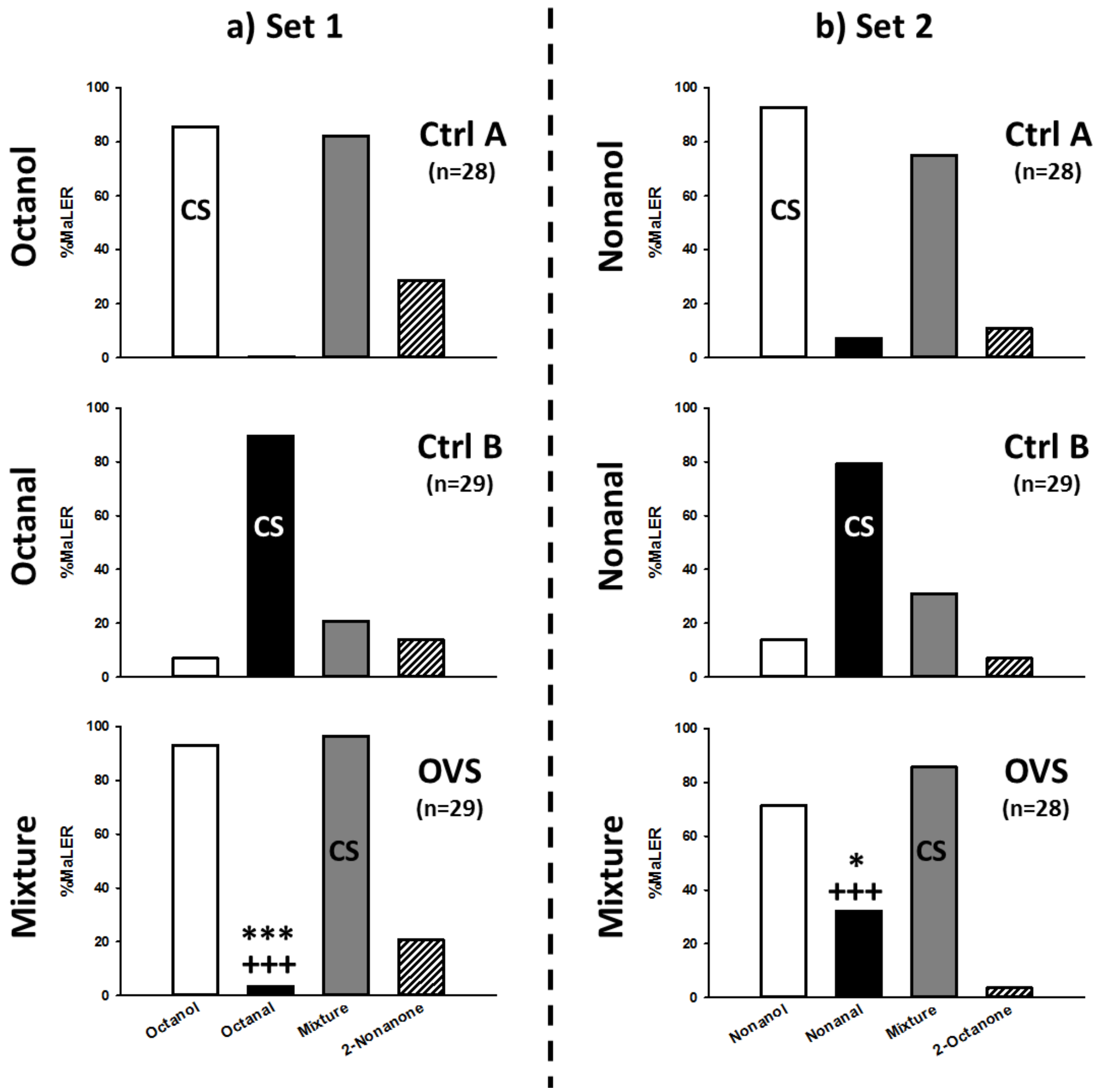

Supplementary Figure 6: **Test responses to combinations of an alcohol and an aldehyde with the same and the longest carbon-chain length of each odour set (set 1: octanol/octanal; set2: nonanol/nonanal). a)** Responses of the control groups (*Ctrl A*: octanol; *Ctrl B*: octanal) and the group trained to the mixture (*OVS*: octanol + octanal). White bars represent responses to octanol, black bars responses to octanal, grey bars responses to the mixture and hatched bars responses to the novel odour tested for generalization (2-nonanone). **b)** Responses of the control groups (*Ctrl A*: nonanol; *Ctrl B*: nonanal) and the group trained to the mixture (*OVS*: nonanol + nonanal). White bars represent responses to nonanol, black bars responses to nonanal, grey bars responses to the mixture and hatched bars responses to the novel odour tested for generalization (2-octanone). Sample size (n) of each group for both odour sets is indicated in parentheses. Overshadowing occurred in both mixtures as shown by the lower responses to one odour after mixture training (white vs. black bar in *OVS* groups: *; $p<0.05$; ***; $p < 0.001$) and by the reduction of responses to that odour after mixture training compared to responses after training to the odour alone (comparison of black bars of *Ctrl B* and *OVS* groups: +++; $p < 0.001$).

Supplementary Figure 7

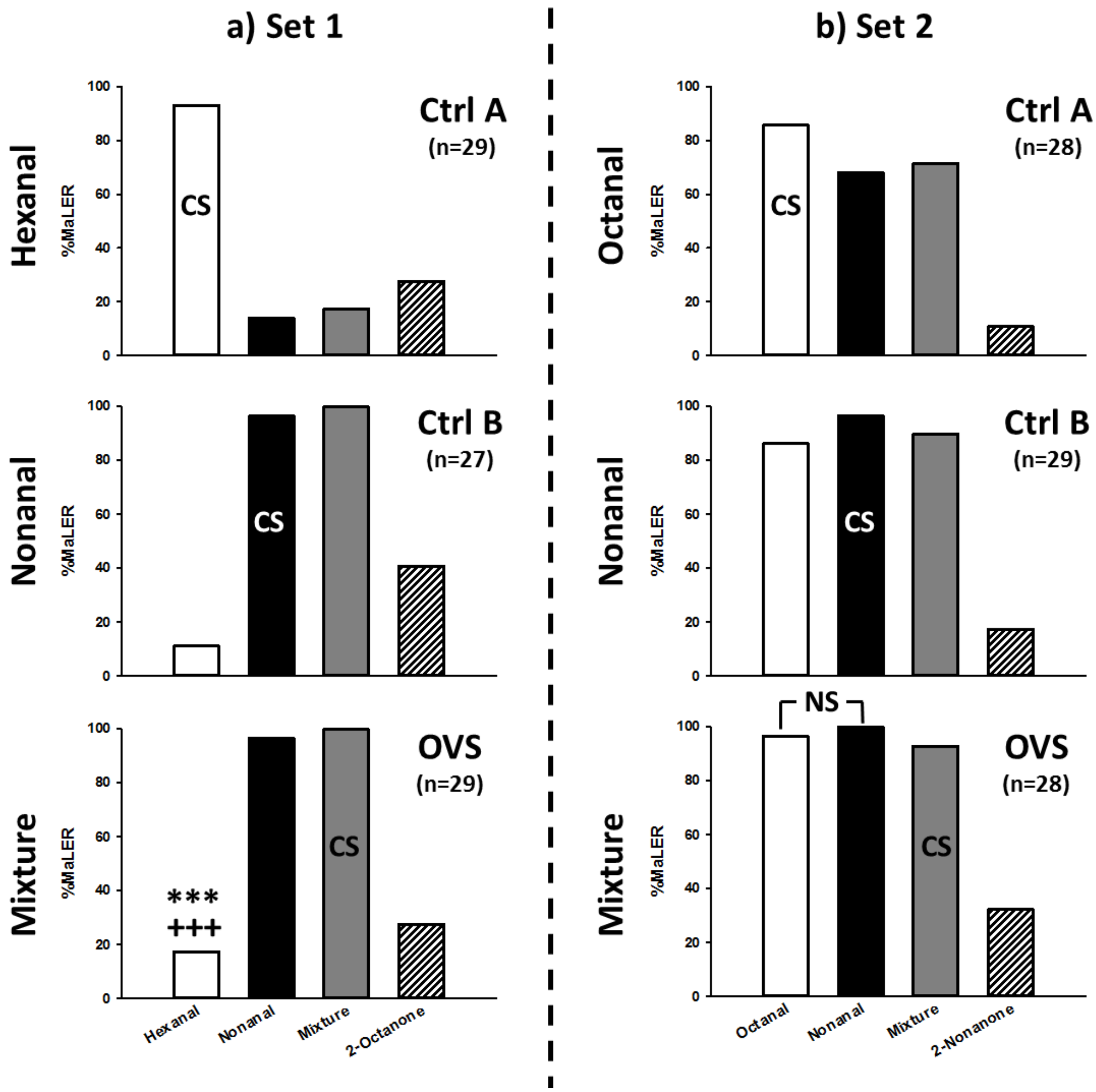

Supplementary Figure 7: Test responses to combinations of two aldehydes with carbon-chain lengths differing in three [a) hexanal/nonanal] or one carbon [b) octanal/nonanal]. a) Responses of the control groups (*Ctrl A*: hexanal; *Ctrl B*: nonanal) and the group trained to the mixture (*OVS*: hexanal + nonanal). White bars represent responses to hexanal, black bars responses to nonanal, grey bars responses to the mixture and hatched bars responses to the novel odour tested for generalization (2-octanone). **b)** Responses of the control groups (*Ctrl A*: octanal; *Ctrl B*: nonanal) and the group trained to the mixture (*OVS*: octanal + nonanal). White bars represent responses to octanal, black bars responses to nonanal, grey bars responses to the mixture and hatched bars responses to the novel odour tested for generalization (2-nonanone). Sample size (n) of each group for both odour sets is indicated in parentheses. Overshadowing occurred in the hexanal + nonanal mixture as shown by the lower responses to hexanal after mixture training (black vs. white bars in the OVS group: ***; $p < 0.001$) and by the reduction of responses to hexanal after mixture training compared to responses after training to the hexanal alone (comparison of white bars between *Ctrl A* and *OVS* groups: +++; $p < 0.001$). The high level of generalization between octanal and nonanal (white and black bars within *Ctrl* A and *Ctrl* B) determined that responses to these odours were

similarly high after mixture training (white and black bars of the *OVS* group). Thus, no overshadowing effect could be detected in this case.

Supplementary Figure 8

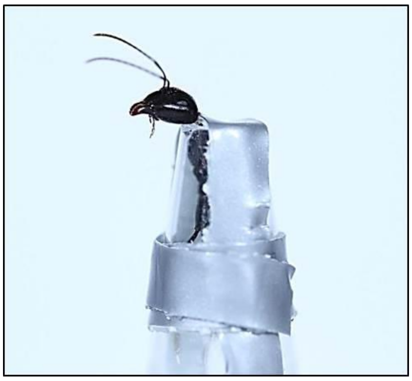

Figure S8: Harnessed *Camponotus aethiops* worker. The ant is harnessed in an individual holder (Eppendorf® tube of 0.2 ml with the tip removed); a strip of adhesive tape is placed between the head and the thorax so that the ant can only move its antennae and mouthparts.
